# Efficacy of resistive exercise on skeletal muscle-related outcomes in cancer survivors: a systematic review protocol

**DOI:** 10.1186/s13643-022-02130-z

**Published:** 2022-11-23

**Authors:** Jacqueline K. Dawson, Dong-Woo Kang, Oscar Barnes, Rebekah L. Wilson, Mary K. Norris, Christina M. Dieli-Conwright

**Affiliations:** 1 Department of Physical Therapy, California State University, Long Beach, Long Beach, CA USA; 2 Division of Population Sciences, Department of Medical Oncology, Dana-Farber Cancer Institute, Boston, MA USA; 3 Department of Medicine, Harvard Medical School, Boston, MA USA; 4 Green Templeton College, University of Oxford, Oxford, UK

**Keywords:** Oncology, Resistance exercise, Muscle performance, Muscle hypertrophy, Muscle-related biomarkers, Cancer patients

## Abstract

**Background:**

Symptom burden and adverse treatment effects can negatively impact physical function, health-related outcomes, and quality of life in cancer survivors. Resistive exercise that improves skeletal muscle function can ameliorate these complications, but the central role of the skeletal muscle in mediating improvements in patient-related outcomes has not been explored. This protocol describes the rationale and methods for a systematic review that aims to determine the effects of resistive exercise on the skeletal muscle hypertrophy, muscle performance, and muscle-related biomarkers in cancer survivors.

**Methods:**

A systematic review will be conducted on peer-reviewed randomized controlled trials (RCTs) that employ resistive exercise interventions for cancer survivors. The following electronic databases will be searched: AMED, CENTRAL, CINAHL, CIRRIE, EMBASE, MEDLINE, PEDro, REHABDATA, Scopus, and SPORTDiscus. Studies will be considered for inclusion if they present quantitative data in adult cancer survivors on skeletal muscle characteristics (e.g., muscle mass), muscle performance (e.g., strength), or skeletal muscle-related biomarkers (e.g., myocellular satellite cells). Secondary outcomes will be physical function (e.g., stair climb) and patient-reported outcomes (e.g., fatigue). Data will be reported through a narrative that describes study design, participants, interventions, and outcome characteristics.

**Discussion:**

This systematic review will help clarify the influence of resistive exercise on factors relating to the skeletal muscle in adult cancer survivors. Findings may provide insight into optimal exercise selection for evidence-based practice.

**Systematic review registration:**

PROSPERO: #277791 [under review]

**Supplementary Information:**

The online version contains supplementary material available at 10.1186/s13643-022-02130-z.

## Background

Cancer incidence and mortality are growing rapidly worldwide, with approximately 19.3 million new cancer cases and nearly 10 million cancer deaths in 2020 according to global cancer estimates (GLOBOCAN) [1]. Although therapeutic advances have significantly improved cancer survival, treatment toxicity and symptom burden persist and can negatively impact physical function, disease-related symptoms, and quality of life [2]. Rehabilitation, especially exercise, is recognized as a key strategy in improving these cancer-related health outcomes and compelling evidence from a 2019 American College of Sports Medicine International Multidisciplinary Roundtable strongly supports the role of exercise in mitigating the adverse effects of treatment across multiple cancer types [3].

The wide therapeutic efficacy of exercise is likely due to its influence on numerous physiological processes, including neuromuscular, cardiovascular, metabolic, and inflammatory systems [4, 5]. Central to mediating the diverse effects of exercise is the skeletal muscle. Decades of research on the role of the skeletal muscle in performance, health, and disease have elucidated functions far beyond force generation and movement [6]. The skeletal muscle is now viewed as an integral component of the complex cross-talk between hormonal, metabolic, and inflammatory pathways in addition to performing its traditional role in the neuromuscular system [7]. Indeed, the influence of the skeletal muscle across multiple systems is apparent in diseases of muscle loss, such as cachexia, and to a lesser extent, sarcopenia and dynapenia, where dysregulation of these diverse pathways can lead to significant morbidity and mortality [7, 8]. Thus, not only is the skeletal muscle essential for physical function, but it is also important for overall health. Furthermore, recent evidence supports the importance of the skeletal muscle in the oncology care continuum as low skeletal muscle mass is associated with higher surgical and postoperative complications, longer length of hospital stay, higher chemotherapy-related toxicity, lower physical function, poorer quality of life, and shorter survival [9, 10].

Previous systematic reviews in the exercise oncology literature involving the skeletal muscle tend to follow a disease-specific (e.g., breast cancer), treatment-specific (e.g., during androgen deprivation therapy), or impairment-specific (e.g., strength loss) approach. For example, systematic reviews by Bourke et al. [11] and Stephensen et al. [12] examined the general benefits of exercise for men with prostate cancer [11] and adults with abdominal cancer [12], while Chen et al. [13] and Hasenoehrl et al. [14, 15] focused specifically on the effect of resistance exercise on physical performance in prostate [13, 14] and breast [15] cancer survivors. On the other hand, some systematic reviews have included all cancer types, but have focused on the type or timing of treatment when evaluating the effects of exercise on physical function [16–18]. One recent review has broadly considered the role of exercise across multiple cancer types, treatments, and impairments to identify key features of exercise interventions that improve physical function and other cancer-related outcomes [19]. This systematic review synthesized data from other exercise oncology systematic reviews with the goal of supporting exercise and rehabilitation intervention decision-making across all cancers. While the aggregate findings identified common features of exercise programs in the general cancer population, a wide range of outcomes were reported.

Hence, to the best of our knowledge, no systematic review has summarized the effects of resistive exercise specific to various skeletal muscle-related outcomes in cancer survivors. Given the significance of the skeletal muscle in physical function and health throughout the oncology care continuum, there is a need to better understand the contribution of the skeletal muscle to physiologic and patient-reported outcomes in cancer survivorship. Therefore, the aim of this systematic review is to comprehensively evaluate the evidence on the effect of resistive exercise on factors relating to the skeletal muscle in adult cancer survivors. The skeletal muscle-related outcomes of interest are primarily focused on muscle mass, performance, and muscle-related biomarkers. The following research questions were formulated to assess this evidence:

1. Which skeletal muscle-related outcomes in cancer survivors are improved by resistive exercise?
2. What are the features of resistive exercise interventions conducted in cancer survivors that demonstrate improvements in skeletal muscle-related outcomes?

## Methods

### Design

This systematic review protocol has been developed according to the Preferred Reporting Items for Systematic Review and Meta-Analysis Protocols (PRISMA-P) [20] (Additional File 1). The protocol is registered in the International Prospective Register of Systematic Reviews (PROSPERO; registration number: 277791).

### Eligibility criteria

We will include studies that meet the following criteria.

#### Study type

Randomized controlled trials (RCTs) or randomized experimental trials written in English that assess the effect of resistive-type exercises on skeletal muscle-related outcomes in adult cancer survivors will be included. Data from reviews, meta-analyses, case reports, or editorials will be excluded.

#### Participants

Adults aged 18 years or older who are diagnosed with cancer regardless of tumor type, stage, or treatment will be included.

#### Intervention

Interventions using any form of resistive exercise are included. Resistive exercise refers to activity involving dynamic (concentric and/or eccentric) or static (isometric) muscle contractions opposed by a force that targets the development or performance of the skeletal muscle. Examples of resistive exercise modalities include free weights, body weight, elastic resistance, plyometrics, constant or variable resistance machines, isokinetic machines, and isometric training, including yoga, Tai Chi, or Pilates. Interventions may use any level of supervision, method of delivery, frequency, duration, or intensity. Multimodal interventions incorporating resistive exercise and other types of intervention, such as aerobic exercise, flexibility training, or diet interventions are not excluded.

#### Comparators

The comparison groups may be a non-resistive exercise group (e.g., aerobic exercise), non-exercise group (e.g., control or waiting list), or standard of care.

#### Outcomes

Studies that report absolute values and/or change from baseline to follow-up on at least one of the following primary outcomes will be considered: (1) hypertrophic characteristics of the skeletal muscle (i.e., muscle mass, cross-sectional area), (2) muscle performance (i.e., strength, muscular endurance, range of motion), or (3) muscle-related biomarkers (i.e., satellite cells, protein synthesis, regulatory gene expression, circulating markers released by muscle). In addition, secondary outcomes of interest will be examined if present alongside a primary outcome. These secondary outcomes include (1) physical function (i.e., stair climb, handgrip strength), (2) patient-reported outcomes (i.e., quality of life, fatigue, pain), or (3) composite scores (i.e., sarcopenia index, frailty index).

### Search strategy

#### Electronic databases

A literature search will be performed in the following databases: Allied and Complementary Medicine Database (AMED), Cochrane Central Register of Controlled Trials (CENTRAL), Cumulative Index to Nursing and Allied Health Literature (CINAHL), Center for International Rehabilitation Research Information and Exchange (CIRRIE), Excerpta Medica Database (EMBASE), Medical Literature Analysis and Retrieval System Online (MEDLINE via PubMed), Physiotherapy Evidence Database (PEDro), National Rehabilitation Information Center Database (REHABDATA), Elsevier Bibliographic Database (Scopus), and EBSCO Sports Medicine Database (SPORTDiscus). The search will be restricted to English language studies, but there will be no restrictions on year of study publication. In addition, we will search the following clinical trial registers to identify ongoing trials: World Health Organization International Clinical Trials Registry Platform (www.who.int/ictrp/en) and ClinicalTrials.gov (www.clinicaltrials.gov).

#### Search terms and keywords

The specific search keywords were developed collaboratively between all authors and included terms from 3 primary domains: (1) cancer (i.e., cancer, tumor, oncology), (2) resistive exercise (i.e., weight lifting, strength training), and (3) muscle outcomes (i.e., hypertrophy, strength, protein synthesis). The MEDLINE search strategy (Table 1) includes a combination of relevant subject headings and keywords. The search strategy for other databases will be adapted from the MEDLINE strategy. Searches will be run with no date limits but within a given time period to ensure consistent data retrieval. The searches will be independently conducted by three review authors (JKD, CDC, MN). Search results will be exported to the systematic review manager Covidence (Melbourne, AU), with duplicate articles removed.

**Table 1:** MEDLINE search strategy

Number	Search items
1	Exp Neoplasms/
2	(cancer* or oncolog* or tumor* or tumour* or neoplas* or carcino* or adenocarcinoma* or metasta*).mp.
3	1 or 2
4	Resistance training/ or resistance exercise/ or weight training/ or plyometric exercise/ or strength training/
5	(weight lifting* or resistance band* or total body* or free weight* or functional move* or hypertrophy training* or repetition maximum* or resistance machine*)
6	Exp Sports/
7	Exp Physical therapy/
8	4 or 5 or 6 or 7
9	3 and 8

^*^Is indicated as a Boolean truncation (or wildcard) operator for database searching, which attaches to the stem of a word and searches for any word that includes that stem or the letters before the asterisk

### Data management

#### Identification and selection of studies

Initial screening of the first 100 articles will be performed by two authors (JKD, CDC) such that each author will examine every record. Any inconsistencies will be discussed until consensus is obtained. These measures are performed for training purposes to ensure the reliability of decision-making by more than one independent reviewer [21]. The two authors who performed the training (JKD, CDC) will then each lead a team of reviewers to independently screen the remainder of the articles. Potentially relevant studies will be identified from titles or abstracts and marked in Covidence as eligible for full-text review. Articles that cannot be safely excluded without reviewing the full text will be included. A second screening will be performed by the two teams of reviewers to assess the full text of eligible articles against the defined criteria for inclusion (Additional File 2). Articles that appear to meet the eligibility criteria will be recorded onto a Google data collection form. One author (JKD) will review all retrieved articles for final inclusion with any disagreements discussed until consensus is reached. Per PRISMA guidelines [22], a flow diagram will be used to describe the process of study selection with reasons for exclusion recorded.

#### Data extraction

Data from each included study will be extracted using predefined criteria that are entered into Covidence to create a data extraction template (Additional File 3). The extracted data include (1) study characteristics: authors, publication year, journal name, country, sample size, and funding source; (2) participant characteristics: inclusion/exclusion criteria, number of participants, age, cancer diagnosis, disease stage, treatment, physical characteristics, and demographics; (3) methods: study design, randomization, blinding, recruitment, retention, and adherence; (4) intervention characteristics: resistive exercise modality, frequency, intensity, session duration, and intervention duration; number of follow-up visits, supervision, progression of intensity, duration, or frequency; (5) primary outcome measures: skeletal muscle characteristics, muscle performance, and myocellular markers; and (6) secondary outcome measures: physical function, patient-reported outcomes, and adverse events. A team of review authors (DK, OB, RW, CC, JY, GC, LM, PM, CH) will independently perform data extraction on all included articles within Covidence. One review author who did not perform the extraction will check the extracted results (JKD).

### Assessment of risk of bias

Methodological quality will be assessed using the risk of bias tool from the Cochrane Handbook for Systematic Reviews of Interventions [23]. The tool has seven domains, including sequence generation, allocation concealment, blinding of participants and personnel, blinding of outcome assessment, incomplete outcome data, selective outcome reporting, and other sources of bias. Each domain will be rated as either high risk, unclear risk, or low risk. One team of authors (DK, OB, RW, CC, JY, GC, LM, PM, CH) will evaluate the risk of bias independently, and differences will be resolved by discussion with an author who did not assess the risk of bias (JKD). Should further clarification of study methods be necessary to assess the risk of bias, we will contact the authors of the study for additional information. The Grades of Recommendation, Assessment, Development, and Evaluation (GRADE) approach will be used to assess the quality of evidence across studies [24].

### Data synthesis

A narrative synthesis of data will be conducted using the Guidance on the Conduct of Narrative Synthesis in Systematic Reviews [25]. The extracted findings will be examined through tables and a descriptive narrative, where data will be grouped according to characteristics to explore patterns between studies. Study quality, strengths, and limitations will be reported. We chose not to pool the data in a meta-analytical approach because of the significant heterogeneity in the reporting of exercise parameters across the different cancer types that would also make it challenging to calculate a valid effect size. Given that our review is the first systematic review on this broad topic, we intend for our descriptive findings to discern patterns that can be targeted in future meta-analytical reviews.

## Discussion

This is the first systematic review to examine the effect of resistive exercise in cancer survivors on skeletal muscle-related outcomes that span multiple domains, including physical performance, muscle characteristics, and tissue level changes. While numerous systematic reviews have been conducted within the exercise oncology literature, prior reviews have focused on specific disease types, such as prostate cancer only, or certain aspects of muscle function, such as strength. Although the consensus is that resistance exercise is beneficial to cancer survivors, limited evidence has been synthesized from RCTs to provide a more holistic view of how the skeletal muscle may respond to resistance exercise to contribute to multiple patient-related outcomes.

This review employs a comprehensive, reproducible approach to searching, screening, and extracting data based on published guidelines and validated methods. Limitations of the review include reporting bias in review authors and significant heterogeneity between studies that may limit the ability to synthesize data. We expect findings from this review to support clinical decision-making by oncology care providers, where evidence from this review may inform exercise selection to improve health-related quality of life in adult cancer survivors.

## Supplementary Information

**Additional file 1.** PRISMA-P Checklist.

**Additional file 2.** Study inclusion spreadsheet.

**Additional file 3.** Data extraction spreadsheet.

## Data Availability

Not applicable

## Abbreviations

AMED: Allied and Complementary Medicine Database
CENTRAL: Cochrane Central Register of Controlled Trials
CINAHL: Cumulative Index to Nursing and Allied Health Literature
CIRRIE: Center for International Rehabilitation Research Information and Exchange
EMBASE: Excerpta Medica Database
GLOBOCAN: Global Cancer Estimates
GRADE: Grades of Recommendation, Assessment, Development and Evaluation
MEDLINE: Medical Literature Analysis and Retrieval System Online
PEDro: Physiotherapy Evidence Database
PRISMA: Preferred Reporting Items for Systematic Review and Meta-Analysis
PRISMA-P: Preferred Reporting Items for Systematic Review and Meta-Analysis Protocols
PROSPERO: Prospective Register of Systematic Reviews
RCT: Randomized controlled trial
REHABDATA: National Rehabilitation Information Center Database
Scopus: Elsevier Bibliographic Database
SPORTDiscus: EBSCO Sports Medicine Database
