# Supplementary material for: Efficacy of resistive exercise on skeletal muscle-related outcomes in cancer survivors: a systematic review protocol
Source: Syst Rev. 2022 Nov 23;11:252. doi: 10.1186/s13643-022-02130-z (PMC9686078; doi:10.1186/s13643-022-02130-z)
Supplement: Supplementary file 2 — Additional file 2. Study inclusion spreadsheet. [file 13643_2022_2130_MOESM2_ESM.pdf]

Article Inclusion Form

Skeletal Muscle and Cancer Review

* Required

1. Reviewer Initials

2. Last name of the first author

3. Year

4. Title

5. Article Type

Mark only one oval.

- ( ) RCT
- ( ) Quasi-RCT
- ( ) Observational
- ( ) Retrospective
- ( ) Longitudinal
- ( ) Protocol Paper
- ( ) Basic science (i.e. non-human study)
- ( ) Review
- ( ) Conference abstract or proceedings only
- ( ) Other: ____________________

6. Cancer Type

____

7. RT used?

Mark only one oval.

- ( ) Yes
- ( ) No

8. List RT modalities (e.g. free weights, plyometrics, etc.)

____

____

____

____

____

9. Which muscle-related outcomes were assessed?

Check all that apply.

- [ ] Muscle mass (e.g. LBM, hypertrophy, CSA, girth, etc.)
- [ ] Muscle performance (e.g. strength, power, etc.)
- [ ] Physical function (e.g. TUG, STS, etc.)
- [ ] Patient-reported outcomes (e.g. fatigue, pain, etc.)
- [ ] Cellular components (e.g. protein synthesis, gene expression)

10. Is study relevant for inclusion? (Human cancer survivors + RT used + at least one muscle-related outcome assessed). *

Mark only one oval.

- ( ) Yes. Upload full-text and mark 'Include' on Covidence.
- ( ) No
- ( ) Don't include but review references

This content is neither created nor endorsed by Google.

Google Forms
