# Supplementary material for: Efficacy of resistive exercise on skeletal muscle-related outcomes in cancer survivors: a systematic review protocol
Source: Syst Rev. 2022 Nov 23;11:252. doi: 10.1186/s13643-022-02130-z (PMC9686078; doi:10.1186/s13643-022-02130-z)
Supplement: Supplementary file 3 — Additional file 3. Data extraction spreadsheet. [file 13643_2022_2130_MOESM3_ESM.docx]

**Additional File 3 - Data Extraction Template**

| **Study Details** | Study ID  Trial registration number  Title  Article title  Funding source or sponsor*  Country or Countries of study completion* |
| --- | --- |
| **Author Details** | Name of first author  Email of corresponding author  Institutions involved |
| **Study Characteristics** | Methods  Study design  Randomized controlled trial (RCT)  Randomized experimental study  Other designs  Single site  Multi-site (2 or more settings)  Trial start date  Trail end date  Defined time-points used in analysis   - T1=baseline, T2 = post-intervention, etc. |
| **Study Setting** | Hospice and/or palliative care unit  Cancer center  Hospital  Community center  Extended care facility  Home-based  University laboratory  Gym and/or wellness center |
| **Study Population** | Participants  Inclusion criteria  Exclusion criteria  Group differences   - Stratification   Total number screened  Total number eligible  Total number enrolled  Total number of withdrawals  Reasons for withdrawal   - Noncompliance - Choose to drop - Lost to follow-up - Disease progression - Death |
| **Study Arms** | Group 1 Name/Name of experimental group  Group 2 Name/Name of comparison group and/or control  Group 3 Name/Name of comparison group  Group 4 Name/ Name of comparison group |
| **Study Arm Details** | Intervention type per group  Total number of participants enrolled to each group  Total number of participants post-tested in each group  Adherence in each group   - Rate (%) or number of sessions completed compared to prescribed |
| **Exercise Prescription** | Frequency   - Number of sessions per week   Intensity   - % Repetition maximum - Rating of perceived exertion - Progression used (Y/N)   Session duration   - Intervention duration   Type   - Free weights - Machines - Body weight - Bands - Plyometric - Aerobic + free weights   Level of supervision (if multiple; described as ‘other’)   - One-on-one - Group - Self-directed - Other |
| **Baseline Characteristics** | Significant differences  Listed characteristics that were significant between groups |
| **Group Descriptives** | Group Name  Number of participants in each group  Age  Gender  Height (cm)  Weight (kg)  BMI (kg/m^2^)  Diagnosis   - Recorded type - Number and/or %   Stage of disease   - Recorded type - Number and/or %   Current treatment   - Recorded type - Number and/or %   Previous treatment   - Recorded type - Number and/or %   Marital status   - Recorded type - Number and/or %   Education level   - Recorded type - Number and/or %   Co-morbidities   - Recorded type - Number and/or %   Medications   - Recorded type - Number and/or %   Physical activity   - Recorded type - Number and/or %   Race   - Recorded type - Number and/or %   Ethnicity   - Recorded type - Number and/or % |
| **Self-Reported Outcome Analysis** | List Patient Reported Instrument Name   - EORTC - FACT-P - Pain - Mood states - Etc.   Report mean change only if provided  Report data at multiple timepoints  Calculate mean change (i.e. T2 – T1 value)  Report by group   - List each instrument - List each subscale - List what timepoint - List each score (mean +/- SD or median [IQR]) - List any between group differences |
| **Objective Outcome Analysis (Muscle Mass)** | List objective outcomes   - Lean body mass (LBM) - DEXA - Cross-sectional area (CSA) - Computed tomography (CT)   Report mean change only if provided  Report data at multiple timepoints  Calculate mean change (i.e. T2 – T1 value)  Report by group   - List each muscle mass measure - List what timepoint - List each value (mean +/- SD or median [IQR])   List any between group differences |
| **Objective Outcome Analysis (Muscle Performance)** | List outcome measures   - Strength (1RM, etc.) - Power (stair climb, etc.)   Report mean change only if provided  Report data at multiple timepoints  Calculate mean change (i.e. T2 – T1 value)  Report by group   - List each muscle performance measure - List what timepoint - List each value (mean +/- SD or median [IQR])   List any between group differences |
| **Objective Outcome Analysis (Physical Function Outcomes)** | List outcome measures   - Timed up and go (TUG) - Sit-to-stand (STS) - Etc.   Report mean change only if provided  Report data at multiple timepoints  Calculate mean change (i.e. T2 – T1 value)  Report by group   - List each physical function performance measure - List what timepoint - List each value (mean +/- SD or median [IQR])   List any between group differences |
| **Objective Outcome Analysis (Composite Outcomes)** | List outcome measures   - Frailty index - Edmonton - Sarcopenia Index - AWGS - Etc.   Report mean change only if provided  Report data at multiple timepoints  Calculate mean change (i.e. T2 – T1 value)  Report by group   - List each combined muscle mass/performance outcome measures - List what timepoint - List each value (mean +/- SD or median [IQR])   List any between group differences |
| **Objective Outcome Analysis (Tissue Outcomes)** | List outcome measures   - Myostatin mRNA expression - Circulating IL-6 - Etc.   Report mean change only if provided  Report data at multiple timepoints  Calculate mean change (i.e. T2 – T1 value)  Report by group   - List each physical function performance measure - List what timepoint - List each value (mean +/- SD or median [IQR])   List any between group differences |
| **Adverse Outcomes** | Adverse event (AE) reporting  AE not recorded by the study  AE recorded, no AE reported for any group  List all AE reported by group   - List type of AE - Number of AE - Timepoint |
